# Likelihood-free Bayesian analysis of neural network models

**DOI:** 10.1186/1471-2202-14-S1-P270

**Published:** 2013-07-08

**Authors:** Brandon M Turner, Per B Sederberg, James L McClelland

**Affiliations:** 1 Psychology Department, Stanford University, Palo Alto, CA, 94035, USA; 2 Psychology Department, The Ohio State University, Columbus, OH, 43210, USA

## 

The goal of cognitive modeling is to understand complex behaviors within a system of mathematically-specified mechanisms or processes; to assess the adequacy of the model to account for experimental data, and to obtain an estimate of the model parameters, which carry valuable information about how the model captures the observed behavior for both individuals and groups. From a theoretical perspective it is essential that we fully understand how the parameters of a model affect the model predictions, and those parameters interact with one another.

Despite the importance of understanding the full range of valid parameter estimates, difficulties encountered in deriving the full likelihood function have prevented the application of fully Bayesian analyses for many cognitive models, especially those that attempt to capture neurally-plausible mechanisms. Recent advances in likelihood-free techniques have allowed for new insights to simulation-based cognitive models [1-3]. Yet, present likelihood-free methods have two critical sources of error that continue to prevent their widespread adoption. The first source of error arises from the use of summary statistics that are not sufficient for the parameters of interest. When a set of summary statistics are not sufficient, one cannot guarantee convergence to the correct posterior distribution. Because it is impossible to guarantee that a summary statistic is sufficient when a likelihood function is unavailable, current likelihood-free estimation techniques introduce error in the posterior distribution, and this error is not directly measurable. The second source of error results from the tolerance threshold that is used to evaluate the approximate likelihood in some algorithms. Even when sufficient statistics are known, a nonzero tolerance threshold will result in inaccurate posterior estimates [2].

Here we present a new, fully-generalizable method, which we call the probability density approximation (PDA) method, for performing likelihood-free Bayesian parameter estimation that does not suffer from these sources of error. Our method works by generating a set of simulated data and constructing an estimate of the underlying probability density function through scaled kernel density estimation. We illustrate the importance of our method by comparing two neural network models of choice reaction time that have never been analyzed using Bayesian techniques due to their computational complexity: the Leaky Competing Accumulator (LCA) [4] model and the Feed-Forward Inhibition (FFI) [5] model. Both models embody neurologically plausible mechanisms such as "leakage", or the passive decay of evidence during a decision, and competition among alternative through either lateral inhibition (in the LCA model) or feed-forward inhibition (in the FFI model). However, it remains unclear as to which dynamical system best accounts for empirical data, due to the limitations imposed by intractable likelihoods. Specifically, complexity measures that take into account posterior uncertainty and model complexity have yet to be applied. Our method of Bayesian analysis leads to results favoring the competitive mechanisms in the LCA over the feed-forward inhibition in the FFI, and reveals parameter trade-offs within these neurologically plausible models as well as interesting individual differences.
